# 3-Iodothyronamine, a Novel Endogenous Modulator of Transient Receptor Potential Melastatin 8?

**DOI:** 10.3389/fendo.2017.00198

**Published:** 2017-08-16

**Authors:** Noushafarin Khajavi, Stefan Mergler, Heike Biebermann

**Affiliations:** ^1^Institute for Experimental Pediatric Endocrinology, Charité University of Medicine Berlin, Berlin, Germany; ^2^Department of Ophthalmology, Charité University of Medicine Berlin, Berlin, Germany

**Keywords:** 3-iodothyronamine, transient receptor potential channel, calcium, thermoregulation, inflammation

## Abstract

The decarboxylated and deiodinated thyroid hormone (TH) derivative, 3-iodothyronamine (3-T_1_AM), is suggested to be involved in energy metabolism and thermoregulation. G protein-coupled receptors (GPCRs) are known as the main targets for 3-T_1_AM; however, transient receptor potential channels (TRPs) were also recently identified as new targets of 3-T_1_AM. This article reviews the current knowledge of a putative novel role of 3-T_1_AM in the modulation of TRPs. Specifically, the TRP melastatin 8 (TRPM8) was identified as a target of 3-T_1_AM in different cell types including neoplastic cells, whereby 3-T_1_AM significantly increased cytosolic Ca^2+^ through TRPM8 activation. Similarly, the β-adrenergic receptor is involved in 3-T_1_AM-induced Ca^2+^ influx. Therefore, it has been suggested that 3-T_1_AM-induced Ca^2+^ mobilization might be due to β-adrenergic receptor/TRPM8 channel interaction, which adds to the complexity of GPCR regulation by TRPs. It has been revealed that TRPM8 activation leads to a decline in TRPV1 activity, which may be of therapeutic benefit in clinical circumstances such as treatment of TRPV1-mediated inflammatory hyperalgesia, colitis, and dry eye syndrome. This review also summarizes the inverse association between changes in TRPM8 and TRPV1 activity after 3-T_1_AM stimulation. This finding prompted further detailed investigations of the interplay between 3-T_1_AM and the GPCR/TRPM8 axis and indicated the probability of additional GPCR/TRP constellations that are modulated by this TH derivative.

## Introduction

Thyronamines (TAMs) are identified as a novel class of endogenous signaling compounds. Currently, two representatives of TAMs, known as 3-iodothyronamine (3-T_1_AM) and thyronamine (T_0_AM), have been identified *in vivo*. Both compounds were detected in blood, heart, brain, thyroid, and many other tissues in rodents (1). Although endogenous TAM concentration may be lower compared to thyroid hormone (TH), it is noteworthy that the tissue-specific and subcellular distributions of TAMs are unknown. Therefore, the concentrations within different cell types might be higher than the average whole tissue concentrations measured.

**Figure 1:**
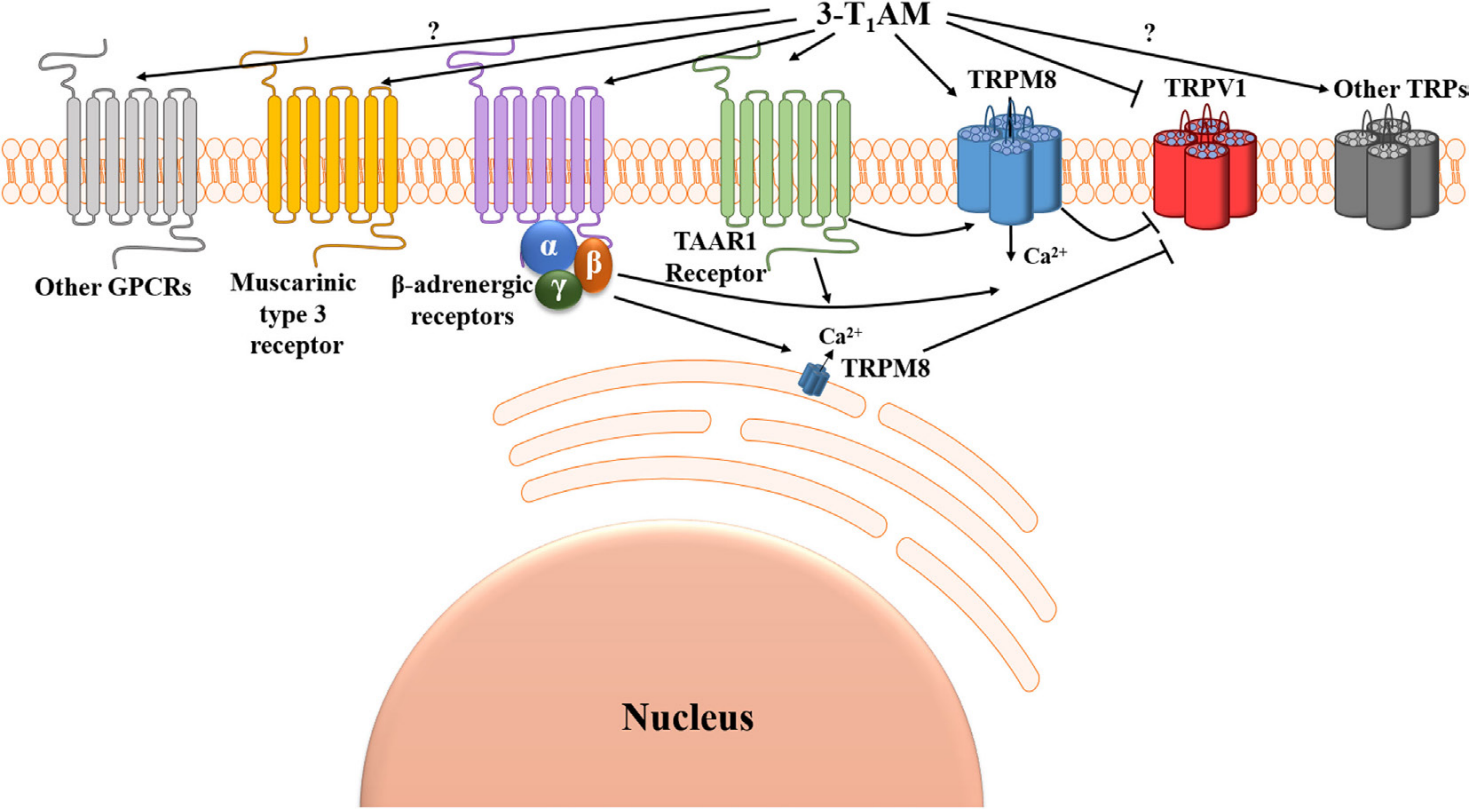
Suggested Ca^2+^ signal transduction pathways induced by 3-iodothyronamine (3-T_1_AM). Two G protein-coupled receptors (GPCRs), known as trace amine-associated receptor 1 (TAAR1) and β-adrenergic receptors, can be activated and one GPCR known as muscarinic type 3 receptor can be suppressed by 3-T_1_AM. 3-T_1_AM increases [Ca^2+^]_i_ *via* a member of the transient receptor channel superfamily known as transient receptor potential channel melastatin 8 (TRPM8) (menthol or cold receptor), and this effect is blocked by BCTC. 3-T_1_AM may either directly activate TRPM8 by a GPCR-independent mechanism or indirectly *via* the β/γ-subunits of Gi/o downstream of β-adrenergic receptors. TRPM8 activation by 3-T_1_AM inhibits TRPV1 (capsaicin or heat receptor)-induced Ca^2+^ influx. Notably, 3-T_1_AM may also directly suppress TRPV1 by a GPCR-independent mechanism (↓[Ca^2+^]_i_). So far, the involvement of other GPCRs and TRPs in 3-T_1_AM-induced signaling effects remained elusive.

3-Iodothyronamine is a decarboxylated and deiodinated TH metabolite (2–4). Administration of 3-T_1_AM in mice resulted in concentration-dependent reversible effects on body temperature, energy metabolism, and cardiac and neurological functions compared with vehicle-treated controls (1). The discovery of 3-T_1_AM and the profound pharmacological effects of this endogenous signaling compound have raised interest to elucidate its signaling properties (1, 3). It is now known that 3-T_1_AM is a “multi-target” ligand, which affects G protein-coupled receptors (GPCRs) and interacts with non-GPCR proteins (5). Classically, the first GPCR-target identified for 3-T_1_AM was a member of the rhodopsin-like family of GPCRs known as trace amine-associated receptor 1 (TAAR1) (1) (Figure 1). Recently, several other GPCRs were identified as targets for 3-T_1_AM, such as α2A adrenergic receptor (6) and β2 adrenergic receptor (7) (Figure 1).

The overall aim of this review is to summarize the modulation of transient receptor potential channels (TRPs) through 3-T_1_AM. Here, we first review the signaling effects of 3-T_1_AM and then focus on TRPs as potential targets for this TH metabolite. In particular, two thermo-TRPs, TRP melastatin 8 (TRPM8) and TRPV, are involved in 3-T_1_AM-induced Ca^2+^ mobilization. Interestingly, both of these TRPs are the key players in thermoregulation and also mediate inflammation in pathophysiological conditions. In this review, we also describe the 3-T_1_AM action on a GPCR/TRP interplay and the possible inverse association between changes in different TRP channel activity modulated by this TH metabolite.

## 3-T_1_AM Declines Body Temperature in Rodents

*In vivo*, 3-T_1_AM partially opposes effects of classical TH action, resulting in a variety of physiological responses (5). Intraperitoneal (ip) injection of 3-T_1_AM blocks the hypothalamic–pituitary–thyroid axis and was shown to reversibly decrease metabolic rate in rodents (1, 8). One of the most prominent effects of 3-T_1_AM in rodents is temporary body temperature decline (1). In clinical settings, this TH metabolite is the only endogenous compound known to induce hypothermia and is additionally suggested to have therapeutic potential for the treatment and prevention of stroke. An ip injection of 3-T_1_AM in adult mice after the experimental induction of stroke led to a reduction of infarct volumes compared with vehicle-treated control mice (9). Due to the profound effects of 3-T_1_AM *in vivo*, an increasing number of studies over the last few years have been devoted to investigate the biosynthetic pathways, functions, and underlying mechanisms behind the effects of 3-T_1_AM.

## Signaling Effects of 3-T_1_AM

Recent studies described the signaling properties of 3-T_1_AM in various cell systems. Interestingly, one study revealed that the functional thyrotropin (TSH)-dependent iodide uptake and TSH-dependent mRNA of sodium/iodide symporter in rat thyrocytes were decreased in the presence of 3-T_1_AM. Therefore, it was congruent to investigate whether 3-T_1_AM modifies signaling pathways downstream of TSH receptor (TSHR) (10). TSH is the major regulator of thyroid function and activation of the TSHR results in Gs as well as Gq signaling in thyrocytes (11, 12). TSH elicits increases of intracellular Ca^2+^ concentration through activation of TSHR-mediated Gq signaling (13). This increase partially occurs through inositol 1,4,5-trisphosphate (IP3)-evoked release of Ca^2+^ sequestered in the endoplasmic reticulum (ER) (14). Previous studies demonstrated the expression of a member of the TRPC family of cation channels known as TRPC2 (pseudogene in human) in rat thyroid cells (15). Surprisingly, reducing the expression of TRPC2 with shRNA decreased Ca^2+^ influx and increased the TSH-induced production of cAMP, which can be due to marked upregulation of TSHR. Nevertheless, this study could not rule out the possibility of Gi signaling inhibition. It has been suggested that deprivation of Ca^2+^ removes the inhibitory action on adenylyl cyclase (AC), upregulates pERK1/2, and increases TSHR expression in thyroid cells (15).

It is known that 3-T_1_AM induces Gs/AC signaling in rat Taar1 and human TAAR1-transfected human embryonic kidney (HEK) cells (1, 16). Recently, it was demonstrated for the first time that 3-T_1_AM increases intracellular Ca^2+^ concentration in rat thyrocytes (PCCL3 cells) (10). TSH-dependent activation of the Gs signaling pathway was not influenced by 3-T_1_AM (10). Furthermore, there is currently no evidence that 3-T_1_AM induced IP3 formation in thyrocytes (10). However, 3-T_1_AM induced increases in cytosolic Ca^2+^ under extracellular Ca^2+^ free conditions in epithelial cells, indicating the intracellular store depletion independent from Gq downstream signaling (17). Therefore, it was concluded that 3-T_1_AM effect in thyrocytes is independent from TSH-induced Gs or Gq signaling (10).

Beside a function of 3-T_1_AM on thyrocytes, it could be shown in another study that 3-T_1_AM enhanced Gs signaling in response to isoprenaline (ISOP) stimulation of the β2-adrenergic receptor in transfected HEK293 cells, but not of β1-adrenergic receptor. Increasing concentrations of 3-T_1_AM in combination with a constant concentration of ISOP modulated Gs-mediated cAMP accumulation. At high 3-T_1_AM concentrations (10^−5^–10^−6^ M), there was a weak increase in ISOP-stimulated cAMP accumulation. In contrast, at lower 3-T_1_AM concentrations (10^−7^–10^−8^ M), a significant increase in ISOP-induced cAMP accumulation was observed, which may be related to the activation of G_i_ signaling (7). These findings in *in vitro* systems indicated that 3-T_1_AM may have a differential impact on certain GPCRs, particularly aminergic receptors, and that the mode of action is concentration dependent. It may also indicate the possibility of biphasic activation (i.e., high and low concentrations may have similar actions, while moderate concentrations enact distinct effects) of different signaling pathways.

Taken together, these studies revealed that 3-T_1_AM enhances GPCR-mediated downstream signaling in different cell types. In addition, β2-adrenergic receptors have been suggested as a new GPCR target for 3-T_1_AM. Nevertheless, the underlying mechanism behind the Ca^2+^ signal transduction remained elusive.

## Ion Channels as Potential Targets for 3-T_1_AM

Although many studies have reported remarkable hypothermia caused by 3-T_1_AM, different observations demonstrated that this effect is not exclusively mediated *via* GPCRs. One study described that 3-T_1_AM-induced decrease of body temperature still persisted in mTaar1 knockout mice (5), which suggested that the actions of 3-T_1_AM are not only mediated *via* the mTaar1 receptor for the induction of hypothermia (18). Recently, it has been shown that hypothermic effects of 3-T_1_AM in mice are due to peripheral vasodilation and subsequent heat loss from the tail surface. Although the possible targets of 3-T_1_AM, Taar1, and the adrenergic receptors were detected in tail arteries and the aorta, neither vessel responded to high doses of 3-T_1_AM. As this anapyrexia effect was also found after intracerebroventricular injection, the authors concluded that this temperature effect might be mediated by non-GPCR targets such as TRPs in the hypothalamus (19). Notably, the systemic temperature lowering effect of 3-T_1_AM lies within the range adequate for eliciting TRP activation. Application of specific blockers as well as overexpression system revealed the involvement of thermo-TRPs in 3-T_1_AM-induced effect. Table 1 summarizes the effects of 3-T_1_AM on Ca^2+^ regulation and whole-cell currents in different cell types. Notably, 3-T_1_AM-induced Ca^2+^ influx varies between normal and neoplastic cells, whereas no differences were detected in whole-cell current densities. This indicates that cytosolic Ca^2+^ regulation induced by 3-T_1_AM might be partially independent from TRP activities. In the following chapter, we summarize the current knowledge about TRP involvement in 3-T_1_AM-induced Ca^2+^ mobilization and downstream signaling.

**Table 1:** 3-T_1_AM effects in different cell types.

[3-T_1_AM] (μM)	Fluorescence ratio (*f*_340 nm_/*f*_380 nm_)	Inward currents (pA/pF)	Outward currents (pA/pF)	Expression localization (cell type)
1.0	↑↑↑↑	N/A	N/A	TRPM8 transfected osteosarcoma [U2OS] (56)
1.0	↑↑↑	N/A	N/A	Thyroid [PCCL3] (10)
1.0	↑↑	−15	108	Human corneal epithelium [HCEC] (56)
1.0	↑↑	−22	161	Human conjunctival epithelium [HCjEC] (17)
1.0	↑	−25	170	Neuroendocrine tumor [BON-1] (unpubl.)
5.0	↑	−25	142	Uveal melanoma [92.1] (unpubl.)

*Fluorescent Ca^2+^ indicator fura-2 is alternately excited at 340 and 380 nm, and the fluorescence ratio (*f*_340 nm_/*f*_380 nm_) is a relative index of changes in [Ca^2+^]_i_*.

*↑ Slight increase, ↑↑ moderate increase, ↑↑↑ strong increase, and ↑↑↑↑ very strong increase*.

*3-T_1_AM, 3-iodothyronamine; TRPM8, transient receptor potential channel melastatin 8*.

### Transient Receptor Potential Channels

Transient receptor potential channels (TRPs) are a superfamily of membrane-spanning non-selective cation channels, which are mainly permeated by Ca^2+^. Classically, TRPs can trigger pain and reception to temperature *via* nociceptors. The TRP superfamily includes 28 members, which can be subdivided into six groups in mammals based on sequence homology (20) and sensitivity to activation by different stimuli: TRPA (ankyrin), TRPC (canonical), TRPM (melastatin), TRPML (mucolipin), TRPP (polycystic), and TRPV (vanilloid) [reviewed in Ref. (21, 22)].

TRPs are located in the plasma membrane and the membrane of various organelles of most cell types. These channels mainly function as homo- and heterotetrameric structures (23, 24). They share the same basic topology, consisting of six transmembrane domains, a pore-forming loop and intracellular N and C termini. In some, but not all subfamilies, the N terminal domain contains ankyrin repeats, which contribute to channel assembly as well as gating and is a common protein–protein interaction motif (25, 26).

TRPs can be activated by thermal, mechanical, or chemical stimuli ranging from ions to small molecules. They are able to integrate and transduce them into appropriate responses in excitable and non-excitable cells (27, 28). There is also some evidence that TRP-elicited responses are modulated by their interactions with other receptors, such as GPCRs or ion channels in various healthy cell types as well as tumor cells (29–31).

### TRP/GPCR Interaction

In mammals, GPCRs and TRPs are coexpressed in a variety of cell types; and different signaling intermediates, such as adaptor proteins, kinases and lipid metabolites, functionally link GPCRs to TRPs (32). TRPs are major downstream effectors of GPCRs, and the signaling pathways that emanate from the activation of GPCRs lead to altered TRP activity or expression (22, 33). Profound understanding of the intracellular Ca^2+^ signaling network, particularly the TRP/GPCR cross-talk and the substantial roles of TRPs, has significantly advanced the field of drug design and development (33, 34). One of the most studied GPCR/TRP regulatory pathways includes the bradykinin receptor (BR), which is coexpressed with TRPV1, TRP ankyrin receptor 1 (TRPA1), TRPM8, and TRPV4 in DRG nerve terminals. Activation of BR leads to rapid stimulation of TRPs to evoke action potentials, resulting in pain and inflammation (32, 35). Another example is coexpression and interaction of muscarinic receptors and TRPV1 in idiopathic overactive bladder urothelial cells, where the cells are responsive to both acetylcholine and capsaicin (36). Interestingly, 3-T_1_AM has been described as a novel antagonist of muscarinic type 3 receptor (37). Although further research on the potential pharmacological effects of 3-T_1_AM in this context is necessary, this interesting example emphasizes the possibility of targeting the TRP/GPCR axis to develop new therapeutic options for different diseases.

## Role of TRPs in Thermoregulation

Six members of the TRP superfamily are recognized as temperature-sensitive TRPs (thermo-TRPs), which are activated at specific temperatures in the range from noxious heat to painful cold (38). Thermo-TRPs are believed to be involved in body temperature perception and based on response patterns can be divided into two subtypes; namely, cold and heat receptors. TRPV1 and TRPV2 respond to painful increases in temperature, while TRPV3 and TRPV4 respond to non-painful increases in temperature. TRPM2 is known as the hypothalamic heat sensor, which mediates the responses to the temperature above 37°C and modulates fever temperature (39). TRPM8 is activated by non-painful decreases in temperature and TRPA1 by painful decrease in temperatures (40, 41). Thermo-TRPs are also substrates of chronic inflammatory mediators released in pathological pain states, which contribute to inflammatory responses and neuropathic pain (42–45). Recent studies demonstrated that TRPM8 and TRPV1 play homeostatic roles in temperature regulation (34).

TRPV1 is the most eminent member of TRP superfamily and has a broad distribution in central and peripheral nervous systems (46). Expression of TRPV1 also has been observed in non-excitable cells (47–49). Generally, TRPV1 can be activated by various stimuli such as heat, vanilloids, cannabinoids, lipids, and protons (50, 51). Administration of TRPV1 agonists triggers both increased heat loss and heat production in mammals (52).

TRP melastatin 8 is found on Aδ and C fiber afferents and is a major determinant of temperature homeostasis including autonomic thermogenesis (30). TRPM8 can be activated by moderate cooling as well as a variety of chemical agonists that are known to produce cool sensations such as menthol and icilin (53–55).

In general, thermo-TRPs can be activated within specific temperature ranges and transduce such inputs into chemical and electrical signals. Different chemical agents are identified which target these channels and elicit the similar downstream effects. So far, 3-T_1_AM is the only known endogenous compound inducing hypothermia and suggested to modulate thermo-TRPs as described in the following paragraph.

## 3-T_1_AM Interacts with Thermo-TRPs

In a recent study, activation of warm-sensitive TRPM2 led to a similar thermoregulatory response observed in mice after systemic administration of 3-T_1_AM (19, 39). An electrophysiological screening of current densities in rat thyrocyte (PCCL3 cells) demonstrated the presence of thermo-TRPs in these cell lines. In PCCL3 cells, 3-T_1_AM induces Ca^2+^ responses similar to specific TRPM8 agonists such as menthol and icilin. Notably, Ca^2+^ elevation was exclusively attenuated in the presence of specific TRPM8 blocker (BCTC) in these cells, which strongly suggests 3-T_1_AM-induced Ca^2+^ rise is attributable to interactions with TRPM8 channels. Recent observations also confirmed the association between TRPM8 and 3-T_1_AM using an osteosarcoma heterologous expression system with overexpressed TRPM8 (56). Furthermore, many studies have demonstrated the endogenous expression of TRPs as well as adrenergic receptors in ocular tissues (17, 56–58). Interestingly, 3-T_1_AM evoked Ca^2+^ mobilization and increases in whole-cell currents in human conjunctival and corneal epithelial cells. This increase in Ca^2+^ influx and in- and outward whole-cell currents were almost fully attenuated in the presence of TRPM8 antagonists (10, 17, 56). Notably, the non-selective adrenergic receptor blocker timolol attenuated 3-T_1_AM-induced Ca^2+^ effects in a similar manner to BCTC, which suggested that 3-T_1_AM activates TRPM8 downstream of GPCRs such as β2 adrenergic receptors (7). Immunostaining pattern indicated TRPM8 expression in ER of ocular cells (17, 56). Previous studies also demonstrated TRPM8 localization in the ER membrane of a prostate cancer-derived epithelial cell line (LNCaP) (59, 60). Consequently, TRPM8 has been suggested as an important ER Ca^2+^ release channel, which is involved in numerous processes in prostate cancer epithelial cells (60). Therefore, the persistence of 3-T_1_AM-induced Ca^2+^ influx in extracellular Ca^2+^ free conditions also might be attributable to intracellular TRPM8 expression in epithelial cells, which supports the hypothesis of 3-T_1_AM-induced Ca^2+^ influx downstream of GPCRs.

Taken together, recent studies suggest that 3-T_1_AM acts as a cooling agent similar to menthol or icilin. It has been proposed that 3-T_1_AM may be a ligand of TRPM8; however, the evidence suggests that its cooling actions are primarily mediated *via* GPCR activation, which indirectly modulates TRPM8 activity. There is accumulating evidence that thermo-TRPs such as TRPM8 and TRPV1 are not only involved in physiological regulations but also a variety of pathophysiological conditions such as inflammation can be influenced by activation of these channels. Here, we describe the role of TRPs in inflammation and the potential of 3-T_1_AM as an anti-inflammatory agent.

## Role of TRPs in Inflammation

Different studies have demonstrated the role of TRPM8 in mediating the anti-inflammatory effects of mild cooling in trauma-induced peripheral inflammation and limiting pain sensation after injury (61, 62). Menthol is one of the most commonly used phytochemical compounds in our daily life due to its analgesic benefit and its ability to provide a cooling sensation (63). Coexpression of TRPV1 with TRPM8 has been demonstrated in many different cell types (64, 65). It is known that multiple inflammatory signaling pathways can be activated downstream of TRPV1 activation by exogenous and endogenous stimuli (66, 67). Capsaicin as a specific TRPV1 agonist elicits increases in pro-inflammatory cytokine release *via* intracellular Ca^2+^ transients, which leads to interleukin secretion (68, 69). The interdependence of TRPM8 and TRPV1 ion channel function has raised interest in the field of anti-inflammatory therapeutic research (70, 71). Previous studies have shown that menthol blocks the mechanical and heat hyperalgesia caused by injection of inflammatory compounds, such as capsaicin (72, 73). Icilin is another specific TRPM8 agonist that is known as a “super-cooling” agent, with a notably higher potency and efficacy than menthol in cellular and behavioral studies (55). Icilin attenuates TRPV1-dependent calcitonin gene-related peptide release in the colon and is a promising therapeutic target for the treatment of colitis (71). Another study also suggested that downregulation of TRPM8 aggravates TRPV1-mediated inflammatory hyperalgesia (70). Although, the recent drug-screening efforts targeting TRPs have resulted in the discovery of effective TRPM8 agonists, the majority of these drugs either were not clinically efficacious or displayed adverse side effects. Subsequently, the general interest for introducing an effective and safe TRP modulator to suppress inflammatory symptoms in different tissues has increased. Recent studies demonstrated that 3-T_1_AM has promising anti-inflammatory cooling properties similar to cooling agents such as icilin (17, 56).

## 3-T_1_AM, a Possible Therapeutic Option for Inflammation?

It has been well-established that TRPM8 activation leads to the suppression of TRPV1 stimulation (70, 71). Thus, we reviewed here the role of 3-T_1_AM in this feedback system. Interestingly, an inverse association between changes in TRPM8 and TRPV1 activity after 3-T_1_AM stimulation has been observed. Specifically, 3-T_1_AM blocked capsaicin-induced TRPV1 activation in human conjunctival and corneal epithelial cell lines and attenuated downstream rises in IL-6 release (17, 56). It was previously described that a TRPV1 antagonist elicited suppression of injury-induced stromal TRPV1 activation in corneal epithelium, which reduced inflammation and fibrosis (74). Notably, the blunting effects of 3-T_1_AM on TRPV1-induced Ca^2+^ influx and IL-6 release mirrored the effects of TRPV1 specific inhibitor (17, 56). This observation revealed a potential therapeutic value of 3-T_1_AM for suppressing TRPV1-induced Ca^2+^ channel-mediated inflammatory processes in different pathophysiological conditions such as dry eye syndrome.

## Conclusion

The TH derivative 3-T_1_AM has been identified as a novel endogenous signaling compound exhibiting remarkable physiological effects such as hypothermia and hyperglycemia, as well as promising therapeutic potential in the experimental prophylaxis and treatment of stroke. Currently, the underlying mechanism of 3-T_1_AM action and its physiological receptor(s) have been insufficiently characterized and are in need of further research. Here, we reviewed the Ca^2+^ signal transduction pathways induced by 3-T_1_AM and provided the promising evidence of TRP channel modulation through this TH metabolite (Figure 1). The 3-T_1_AM action on GPCRs as well as on TRPs indicates the complex functional (co)-regulation of each system, which have a high impact on physiological and pathophysiological conditions. The close crosstalk of GPCRs and TRPs provides the opportunity to widen the options for therapeutic intervention, and by using such coregulated systems the possibility of unwanted side effects might be reduced.

## Author Contributions

NK and HB contributed to the conception, design, and drafting of this review. NK created the figure. SM created the table and contributed to the conception of this review. All the authors read and approved the final manuscript.

## Conflict of Interest Statement

The authors declare that the research was conducted in the absence of any commercial or financial relationships that could be construed as a potential conflict of interest.
